# A Grandparent in the room: multiple family therapy for adolescents

**DOI:** 10.1186/2050-2974-2-S1-P13

**Published:** 2014-11-24

**Authors:** Carol Smith, Julie Potts, Kimberly Hoiles, Uli O'Sullivan

**Affiliations:** 1 Eating Disorders Program, Specialised Child and Adolescent Mental Health Service, Department of Health in Western Australia, Perth, Australia

## Objective

To provide a descriptive clinical perspective on the introduction and development of Multiple Family Therapy Groups for Day Patients and Outpatients at Princess Margaret Hospital, Perth Western Australia. Challenges will be discussed including motivation, integration and application with view to sharing with the audience the pitfalls and possibilities of this dynamic approach combining family and group therapies.

## Method

Retrospective clinical perspectives, a composite case and literature review will be presented to highlight the exploration and evolution of a multiple family therapy groups.

## Discussion

The focus of discussion will be on lessons learnt about the process of adapting clinical treatments within existing service frameworks including strategy for motivating and engaging both staff and patients in the process of change.

## Findings

With creativity and collaboration, multiple family therapies can be integrated within existing treatment frameworks, research grants can facilitate the process of program development and extended families can become important treatment allies.

## Conclusion

Multiple family therapy can be adapted to fit existing service frameworks and offers new possibilities for engagement with the adolescent and their wider family system. Grandparents, siblings, uncles and aunties are all resources in the treatment of an adolescent with an eating disorder, and can offer alternative portals of hope and support in the recovery process.

